# Hyperreflective foci in predicting the treatment outcomes of diabetic macular oedema after anti-vascular endothelial growth factor therapy

**DOI:** 10.1038/s41598-021-84553-7

**Published:** 2021-03-03

**Authors:** Chu-Hsuan Huang, Chang-Hao Yang, Yi-Ting Hsieh, Chung-May Yang, Tzyy-Chang Ho, Tso-Ting Lai

**Affiliations:** 1 Department of Ophthalmology, Cathay General Hospital, Taipei, Taiwan; 2 Department of Ophthalmology, National Taiwan University Hospital, No 7, Chung-Shan S. Rd., Taipei, 100 Taiwan; 3 Department of Ophthalmology, College of Medicine, National Taiwan University, Taipei, Taiwan; 4 Graduate Institute of Clinical Medicine, College of Medicine, National Taiwan University, No 7, Chung-Shan S. Rd., Taipei, 100 Taiwan

**Keywords:** Retinal diseases, Prognostic markers

## Abstract

This retrospective study evaluated the association of hyperreflective foci (HRF) with treatment response in diabetic macular oedema (DME) after anti-vascular endothelial growth factor (VEGF) therapy. The medical records, including of ophthalmologic examinations and optical coherence tomography (OCT) images, of 106 patients with DME treated with either intravitreal ranibizumab or aflibercept were reviewed. The correlations between best-corrected visual acuity (BCVA) changes and HRF along with other OCT biomarkers were analysed. The mean logMAR BCVA improved from 0.696 to 0.461 after an average of 6.2 injections in 1 year under real-world conditions. Greater visual-acuity gain was noted in patients with a greater number of HRF in the outer retina at baseline (p = 0.037), along with other factors such as poor baseline vision (p < 0.001), absence of epiretinal membrane (p = 0.048), and presence of subretinal fluid at baseline (p = 0.001). The number of HRF after treatment was correlated with the presence of hard exudate (p < 0.001) and baseline haemoglobin A1C (p = 0.001). Patients with proliferative diabetic retinopathy had greater HRF reduction after treatment (p = 0.018). The number of HRF in the outer retina, in addition to other baseline OCT biomarkers, could be used to predict the treatment response in DME after anti-VEGF treatment.

## Introduction

Diabetic macular oedema (DME), occurring in 3–9% of patients with diabetes, is a sight-threatening condition arising in cases of diabetic retinopathy^1–4^. Among the different treatments for DME, anti-vascular endothelial growth factor (anti-VEGF) therapy, compared with laser photocoagulation and steroid treatment, has been shown to lead to superior visual outcomes^5^. Furthermore, intravitreal injection (IVI) of anti-VEGF agents such as ranibizumab and aflibercept has been shown to significantly improve the vision and macular anatomy of patients with DME in clinical trials, especially when injections were administered under strict loading and re-treatment protocols^6–8^. However, the frequency of re-treatment in the real world was often lower than that recommended, therefore resulting in relatively inferior outcomes compared with those of the clinical trials^9,10^.

The treatment response of DME after anti-VEGF injection differs between clinical trial and real-world studies and varies among patients. To better understand the prognosis of DME after anti-VEGF therapy, several clinical biomarkers have been correlated with treatment outcomes, including central foveal thickness (CFT), external limiting membrane disruption, ellipsoid zone disruption, subretinal fluid (SRF), and presence of hyperreflective foci (HRF)^11–13^. Bolz et al. firstly reported the presence of HRF and their characteristics on optical coherence tomography (OCT) in patients with DME^14^, but their nature remained unclear despite several possible origins having been proposed, including lipid extravasation from a compromised vasculature, microglia proliferation, or retinal pigmented epithelium (RPE) migration^14,15^. In addition, previous studies have also reported controversial results regarding the correlation between HRF and visual acuity, especially that the presence of HRF in different retinal layers at baseline might impact visual improvement after anti-VEGF therapy differently^16–20^. Therefore, unlike other OCT biomarkers such as SRF, which have been determined to predict the treatment response of DME^12,21^, the role of HRF as a predicting factor for DME has not been determined.

The purpose of this study was to investigate the treatment response of DME and its correlation with OCT biomarkers, especially the presence of HRF in different retinal layers.

## Results

### Visual and anatomical outcomes

**Figure 1:**
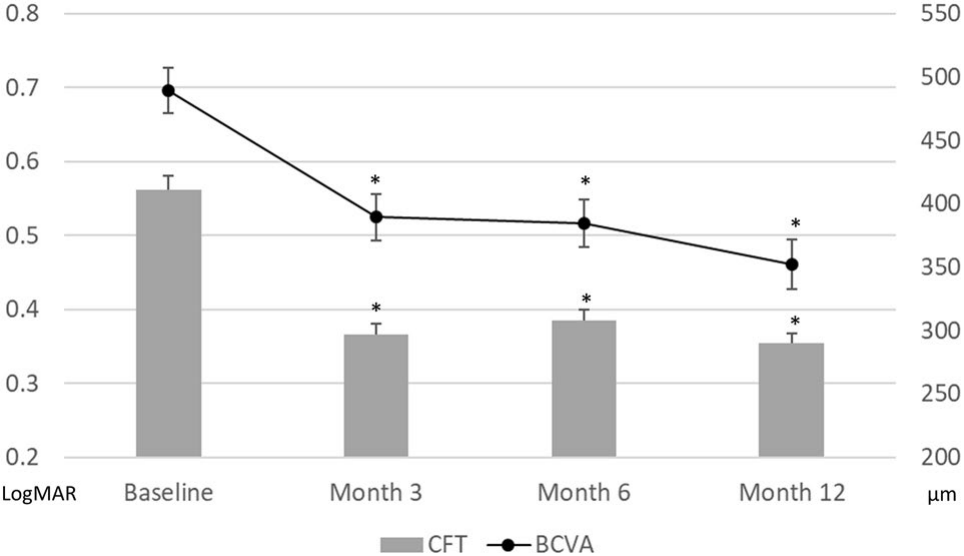
The average BCVA and CFT at the baseline and 3, 6, 12 months after anti-VEGF treatment for diabetic macular oedema. Both BCVA and CFT significantly improved compared with baseline at 3, 6, and 12 months after initial anti-VEGF treatment. *BCVA* best-corrected visual acuity, *CFT* central foveal thickness, *VEGF* vascular endothelial growth factor. *p < 0.001 (compared with baseline).

The demographic data of the 106 patients with diabetic macular oedema who were enrolled are summarised in Table 1. Overall, the average age was 62.7 ± 9.2 years, and 45.3% of the patients were male. Proliferative diabetic retinopathy (PDR) was confirmed by fluorescein angiography in 31.4% of patients. There were 69.8% treatment-naïve patients in our cohort. The mean logarithm of the minimum angle of resolution (logMAR) best-corrected visual acuity (BCVA) at baseline was 0.696 ± 0.317. Factors associated with poor baseline vision included thicker CFT, presence of SRF, and a greater number of HRF in the inner retina (p < 0.001, p = 0.005, p = 0.014, respectively). The average BCVA at 3, 6, and 12 months after the first injection was 0.525 ± 0.323, 0.516 ± 0.330, and 0.461 ± 0.351, respectively (Fig. 1), and significant visual improvements were found at every time point compared with baseline (all p < 0.001). The mean injection numbers were 4.2 ± 1.0 in the first 6 months and 6.2 ± 2.1 in 1 year.

**Table 1:** Demographic data at the baseline of the patients who received anti-vascular endothelial growth factor treatment for diabetic macular oedema.

**Baseline characteristics (N = 106)**	
Age (y/o)	62.68 ± 9.21
Sex (male)	45.28%
HbA1C	7.63 ± 1.13%
Hypertension	62.22%
Pseudophakic	29.24%
Previous treatment	30.19%
PDR	31.43%
Hard exudate	45.28%
Baseline VA (LogMAR)	0.696 ± 0.317
Baseline CFT (μm)	410.86 ± 115.33
Epiretinal membrane	31.13%
Intraretinal cyst	86.79%
Subretinal fluid	31.13%

*HbA1C* haemoglobin A1C, *PDR* proliferative diabetic retinopathy, *VA* visual acuity, *CFT* central foveal thickness.

### Association of hyperreflective foci with visual and anatomical improvements at 1 year

A greater number of HRF in the outer retina at baseline was correlated with better BCVA improvement at the 12-month follow-up (p = 0.037). However, the number of HRF in the inner retina was not associated with BCVA improvement. Other factors correlated with BCVA improvement are listed in Table 2. Patients with history of hypertension, previous treatment for DME, and presence of epiretinal membrane (ERM) at baseline had lesser visual improvement after treatment (p = 0.001, 0.031, and 0.048, respectively). In contrast, worse baseline vision and the presence of SRF at baseline were correlated with better BCVA improvement (p < 0.001 and p = 0.001, respectively). As for the treatment response of CFT, a greater number of HRF in both the inner and outer retina at baseline was correlated with greater CFT improvement at the 12-month follow-up (see Supplementary Table S1 online).

**Table 2:** Baseline factors associated with best-corrected visual acuity improvement at 1 year in patients with diabetic macular oedema under anti-vascular endothelial growth factor therapy.

	VA change at the 12th month	
	P-value	Coefficient
Hypertension	0.001	0.209
Previous treatment	0.031	0.136
Baseline VA (LogMAR)	< 0.001	− 0.337
Baseline epiretinal membrane	0.048	0.123
Baseline subretinal fluid	0.001	− 0.209
Baseline HRF in the outer retina	0.037	− 0.007

*VA* visual acuity, *HRF* hyperreflective foci.

### Literature review regarding the role of hyperreflective foci in predicting visual outcomes

We identified 13 studies that reported on the association between HRF and final visual acuities or visual improvements^16–19,22–30^. The differences in study designs and their results are summarised in Table 3. Two studies reported greater VA improvement in the presence of HRF; one of them found that the correlation was limited to HRF in the outer retina, and the other found a correlation between VA gain and HRF in both the inner and outer retina. Three studies reported no association between HRF and final VA or VA improvement, and the other 8 studies reported less VA improvement or inferior final VA in the presence of baseline HRF.

**Table 3:** Studies evaluating the role of baseline hyperreflective foci in predicting visual outcomes after treatment for diabetic macula oedema.

Study	Design	N	Treatment	F/u (m)	Assessment	Layering	VA implications
**Associated with better VA outcome**							
Current study	Retro	106	Ranibizumab/Aflibercept	12	Quantitative	Inner/outer/SRF	Amounts of HRF in the outer retina associated with greater VA improvement
Schreur et al.^19^, 2018	Retro	54	Bevacizumab	3	Quantitative	Inner/outer	Amounts of HRF associated with greater VA improvement
Yoshitake et al.^29^, 2020	Retro	77	Ranibizumab	12	Qualitative	Inner/outer	Presence of HRF in the outer retina associated with greater VA improvement
**No association with VA outcome**							
Framme et al.^16^, 2012	Retro	51	Ranibizumab Bevacizumab	1	Quantitative	Whole retina	Amounts of HRF not associated with final VA
Vujosevic et al.^27^, 2016	Pro	20	Ranibizumab	6	Quantitative	3 layers	Amounts of HRF not associated with final VA
Ahn et al.^22^, 2020	Retro	45	Dexa implant Anti-VEGF	1	Quantitative	Within IRC	Amounts of HRF within IRC not associated with VA improvement
**Associated with worse VA outcome—poor VA improvement**							
Zur et al.^30^, 2018	Retro	299	Dexa implant	4	Quantitative	Inner/outer	Presence of HRF associated with poor VA improvement
Murakami et al.^25^, 2018	Retro	23	Ranibizumab	3	Qualitative	Within IRC	Presence of HRF in IRC associated with poor VA improvement
**Associated with worse VA outcome—poor final VA**							
Nishijima et al.^26^, 2014	Retro	32	Vitrectomy	Mean: 15.3	Qualitative	Outer	Presence of HRF in the outer retina associated with poor final VA
Kang et al.^17^, 2016	Retro	97	Bevacizumab	Mean: 6.71	Quantitative	Inner/outer	Amounts of HRF in the outer retina associated with poor final VA
Chatziralli et al.^24^, 2016^a^	Retro	92	Ranibizumab Dexa implant	9	Quantitative	Whole retina	Amounts of HRF associated with poor final VA
Chatziralli et al.^23^, 2017^b^	Pro	54	Dexa implant	12	Qualitative	Whole retina	Presence of HRF associated with poor final VA
Weingessel et al.^28^, 2018	Pro	50	Ranibizumab + laser PRN	60	Qualitative	Whole retina	Presence of HRF cluster associated with poor final VA
Liu et al.^18^, 2019	Retro	26	Conbercept	3	Quantitative	Inner/outer/sub-RPE	Amounts of HRF associated with poor final VA

*N* case number, *F/u* follow-up interval (months), *VA* visual acuity, *Retro* retrospective, *Pro* prospective, *IRC* intraretinal cyst, *Dexa implant* dexamethasone implant, *HRF* hyperreflective foci, *SRF* subretinal fluid, *VEGF* vascular endothelial growth factor, *RPE* retinal pigment epithelium, *PRN* pro re nata.

^a^This study included macula oedema resulted from diabetic retinopathy and retinal vein occlusion.

^b^This study included diabetic macular oedema patients refractory to anti-VEGF treatment.

### Factors associated with the amount of hyperreflective foci

**Figure 2:**
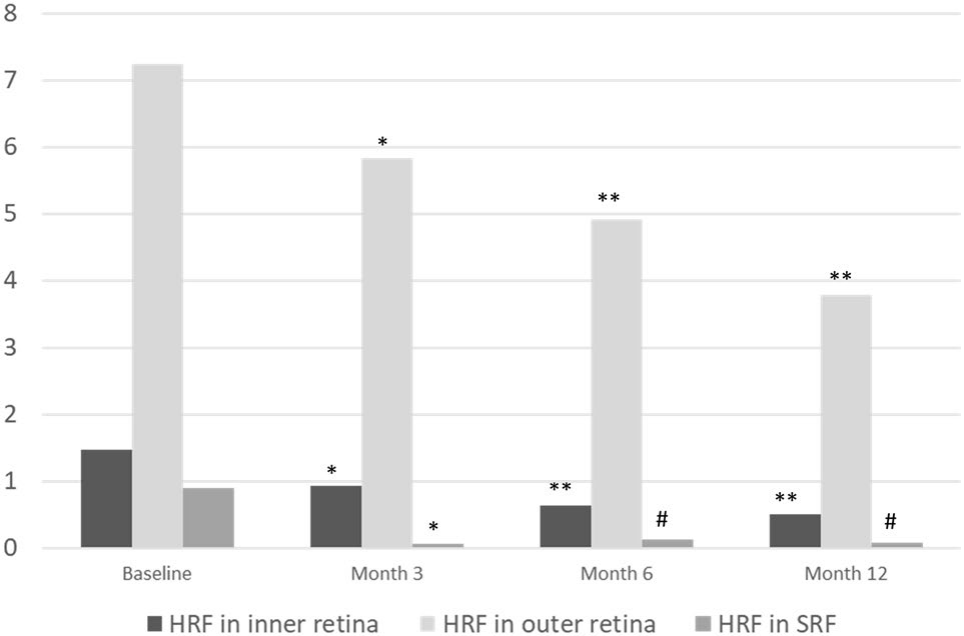
The average number of HRF in different retinal layers at the baseline and 3, 6, 12 months after anti-vascular endothelial growth factor treatment for diabetic macular oedema. The number of HRF in every retinal layer significantly decreased in all follow up time points compared with baseline. *HRF* hyperreflective foci, *SRF* subretinal fluid. *p = 0.001 (compared with baseline). ^#^p = 0.002 (compared with baseline). **p < 0.001 (compared with baseline).

The number of HRF at different anatomical locations and different time points (Fig. 2) was correlated with baseline HRF amounts and other demographic data (Table 4). The presence of hard exudate correlated with the number of HRF at every time point and every layer of the retina (p < 0.001 for all). The number of HRF in the inner and outer retina after treatment was positively correlated with haemoglobin A1C (HbA1c) (p = 0.002 and 0.001, respectively). The number of HRF in the outer retina was more greatly reduced after treatment in younger patients (p = 0.037) and in patients with PDR (p = 0.018).

**Table 4:** The coefficient and P-value in the linear regression model for possible associated baseline characteristics of the number of HRF in the inner and outer retina at different time points.

	HRF in the inner retina, P-value (coefficient)			HRF in the outer retina, P-value (coefficient)		
	Baseline	6th month	12th month	Baseline	6th month	12th month
Age	0.896	0.517	0.886	0.157	0.027 (0.086)	0.037 (0.087)
Sex	0.217	0.157	0.394	0.682	0.146	0.187
HbA1c	0.984	0.024 (0.198)	0.002 (0.280)	0.632	0.001 (1.053)	0.001 (1.108)
HTN	0.545	0.451	0.363	0.883	0.325	0.362
Naïve	0.26	0.739	0.793	0.132	0.447	0.384
PDR	0.862	0.989	0.057	0.579	0.010 (− 2.014)	0.018 (− 2.047)
HE	< 0.001 (1.665)	< 0.001 (0.588)	< 0.001 (0.744)	< 0.001 (9.794)	< 0.001 (5.374)	< 0.001 (5.851)

The coefficient of significant factors (p-value < 0.05) is shown within the brackets.

*HbA1C* haemoglobin A1C, *HTN* hypertension, *PDR* proliferative diabetic retinopathy, *HE* hard exudate.

### Comparison of the treatment outcomes of different anti-VEGF agents

Among all 106 cases enrolled, 65 eyes received IVI treatment with ranibizumab and 41 eyes with aflibercept. There was no difference between the groups at baseline except for more ERM in the aflibercept group (15 [23%] vs. 18 [44%], p = 0.024) and a higher incidence of SRF in the ranibizumab group (25 [39%] vs. 8 [20%], p = 0.04). The two groups had comparable visual improvements at every time point. There were slightly fewer total injections in the aflibercept group than in the ranibizumab group (6.5 ± 2.1 vs. 5.7 ± 1.9) with borderline significance (p = 0.080). There was no interaction between the amounts of HRF in the outer retina and the drug type used in the regression model in predicting VA improvement (p = 0.207).

## Discussion

In this present study, we demonstrated the efficacy of anti-VEGF therapy in treating DME, both anatomically and functionally, under real-world conditions. The treatment effect was sustained during the 1-year follow-up with an average of six injections. In addition, a greater number of HRF in the outer retina at baseline predicted better visual improvement, as did other OCT biomarkers such as absence of ERM and presence of SRF.

Bolz et al. first introduced the characteristics and localisation of HRF found on OCT among patients with DME^14^. These HRF were commonly distributed through the retinal layers, including the subretinal space. In addition to their presence in DME and advanced DR, HRF were also found in the early stage of DR with inadequate glucose and blood pressure control^31^. The different origins of HRF, including extravasated lipids from compromised microvessels in diabetic retinopathy^14^, microglia activated by inflammation^32^, and migrating RPE cells^33^, might contribute to the wide distribution of HRF observed in our study.

Despite their undetermined nature, HRF have been linked to visual outcome, yet the results varied among different studies (Table 3). Uji et al. found that the presence of HRF in the outer retina was related to poor baseline vision and disrupted anatomical structure in DME before treatment^20^. In the present study, we found that patients with a higher number of HRF in the outer retina had enhanced visual improvement at the 1-year follow-up, which was in accordance with the findings of two recent reports^19,29^. Other studies have reported inferior visual outcome or limited improvement after anti-VEGF therapy with the presence of HRF at baseline^17,18,23–26,28,30^. The inconsistency might have resulted from the wide range of HRF present at baseline across studies. Furthermore, the HRF in different studies may have had different origins, thus showing different predictive values. In addition, not every study separately evaluated HRF in different retinal layers. In our study, we evaluated HRF according to their presence in different retinal layers; hence, the exact role of HRF in outcome prediction could be better appreciated.

To date, there is limited literature focusing on the relationship between other baseline biomarkers and the presence of HRF before and after treatment. In the current study, we noted a strong correlation between the presence of hard exudate at baseline and the presence of HRF in every layer before and after treatment. This finding supported the hypothesis that HRF represents lipid extravasation as subclinical hard exudate. Furthermore, patients with PDR at baseline exhibited fewer residual HRF in the outer layer after treatment. The high concentration of VEGF in patients with PDR possibly caused increased vascular permeability and leakage, which further formed HRF. As a result, the HRF in these eyes had a better response under adequate anti-VEGF therapy, and thus fewer HRF after treatment. The level of HbA1c, representing the average status of blood glucose control, was reported to be independent of the prognosis of DME treatment in a large clinical trial^34^. However, in a real-world retrospective study, better HbA1c was correlated with superior visual and anatomical outcomes^35^. Framme et al. also showed that a greater number of HRF was found in patients with higher HbA1c^16^. In this study, higher HbA1C at the time of diagnosing DME, possibly representing a more ischaemic retinal environment, was related with a greater number of HRF in the inner and outer retina after anti-VEGF therapy. This effect lasted for 12 months after first injection.

A previous study linked the presence of HRF to a higher grade of inflammation, which might contribute to poor response to anti-VEGF therapy^32^. Nevertheless, Chatziralli et al. demonstrated that both intravitreal anti-VEGF and steroid therapy could result in a similar degree of HRF reduction in retinal vascular disease, along with comparable visual outcomes^24^. Our study also found significantly decreased HRF after anti-VEGF therapy, and the reduction of HRF was similar among eyes treated with different anti-VEGF agents. The efficacy of different anti-VEGF agents in treating DME has been evaluated in previous studies and showed conflicting results. While one study with a small number of cases reported similar efficacy between aflibercept and ranibizumab^36^, other studies showed superior efficacy of aflibercept^37,38^. Moreover, in DRCR.net protocol T, they reported a better visual gain with aflibercept compared to ranibizumab at 1 year, especially in patient with worse baseline vision (worse than 20/50)^39–41^. However, the difference was not significant at the 2-year follow-up^40^. In our study, which only included patients with baseline vision worse than 20/50, the visual improvements were comparable in both groups in a clinical practice setting, with a 12.5-letter gain in the ranibizumab group and a 10-letter gain in the aflibercept group, which were both inferior to the results of the clinical trial. In addition, different anti-VEGF agents did not affect the predictive value of HRF in our study.

Other biomarkers were also reported to be associated with VA outcomes. The presence of SRF was correlated with better visual acuity improvement and CFT reduction in our study, which were in accordance with previous findings^42,43^, especially when the patients had been treated aggressively^21^. Conversely, the presence of ERM contributed to inferior functional and anatomical outcomes^44^. In addition to those baseline OCT biomarkers, the BCVA response at 3 months also independently predicted the visual outcome at 12 months (see Supplementary Table S1 online) as described in a previous study^45^.

The major limitation of this study was its retrospective nature and the relatively small number of patients. Furthermore, the decision and timing for re-injection were also based on variable clinical conditions and decided by the patient and physician. However, our results constitute evidence derived from a real-world setting and provide additional information, complementing the findings of clinical trials, to help guide physicians in their routine clinical practice. Another limitation was the possibility of selection bias, especially when comparing the treatment outcomes between different anti-VEGF agents. Although most baseline factors were balanced, the prevalence of ERM and SRF was slightly different at baseline between the aflibercept group and the ranibizumab group.

In conclusion, we reported our real-world experience with the 1-year outcome of anti-VEGF treatment for DME, showing significant visual and anatomical improvements with both aflibercept and ranibizumab. In addition, we demonstrated that OCT biomarkers including SRF, ERM, and HRF in the outer retinal layer could help predict the treatment outcomes in patients with DME after anti-VEGF therapy. Furthermore, the number of HRF was associated with the presence of hard exudates at baseline, the status of diabetic retinopathy, and blood glucose control. Our results could help predict the treatment response in patients with DME and might further facilitate individualised treatment.

## Methods

### Study population and intervention

We retrospectively reviewed the records of patients who had been diagnosed with DME and treated with 0.5 mg of ranibizumab or 2 mg of aflibercept in National Taiwan University Hospital from January 2016 to December 2017. The study has been approved by the Institutional Review Board of National Taiwan University Hospital and was performed in accordance with the guidelines and regulations of the Institutional Review Board; the study also adhered to the tenets of the Declaration of Helsinki. The necessary informed consent was obtained from all patients. Patients with OCT- and fluorescein angiography-evident central involving DME and a baseline BCVA between 20/400 and 20/40 (Snellen) were included in this study. All patients were treated with two or three monthly loading injections, followed by as needed injections according to the BCVA change and OCT findings at each follow-up visit. Each patient visited the clinic every 4–8 weeks for at least 1 year. The choice between ranibizumab and aflibercept was based on the physician’s preference. The exclusion criteria included a serum haemoglobin A1c (HbA1c) level greater than 10% at baseline, a history of receiving vitrectomy, glaucoma, previous treatments for DME (e.g. macular laser, intravitreal steroid, and anti-VEGF agents) within 6 months prior to the first IVI in our hospital, presence of macular scar, less than two loading injections of anti-VEGF, and presence of coexisting retinal vascular disease.

### Data collection

**Figure 3:**
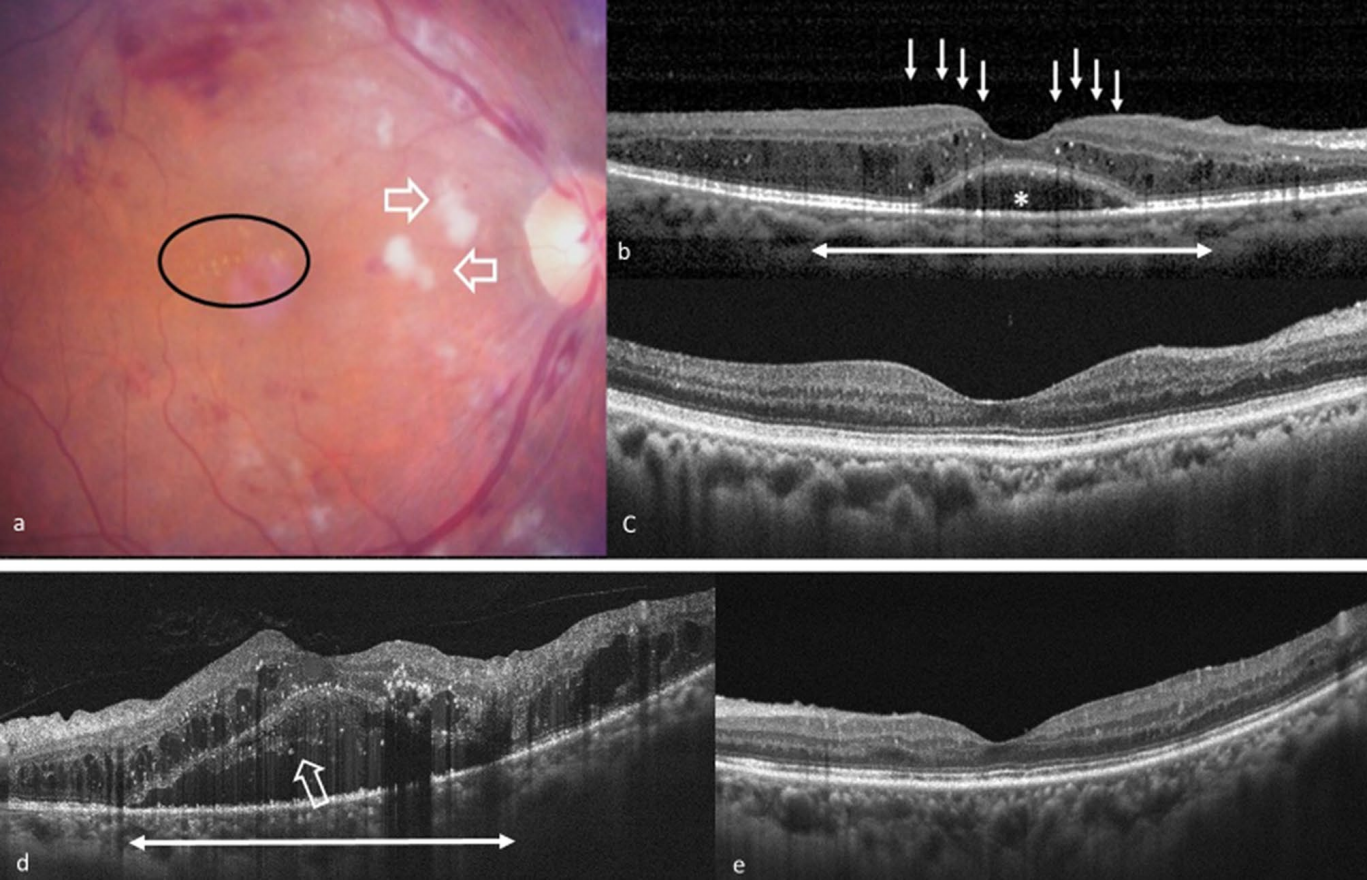
Two representative cases of patients who had DME and underwent anti-vascular endothelial growth factor treatment. (**a**–**c**) A 67-year-old female patient without previous treatment for DME. (**a**) The fundus photography at the baseline revealed severe non-proliferative diabetic retinopathy with diffuse retinal haemorrhage and cotton wool spots (empty arrows). There was a limited amount of hard exudate in the macular area (circle). (**b**) The OCT at baseline exhibited DME and the presence of SRF (asterisk). Large numbers of HRF, mostly located in the outer retina (white arrows), were found within 3 mm of the central macula (double arrow). The baseline visual acuity was 20/100, and CFT was 406 μm. (**c**) The patient received three injections in 1 year, and the final OCT revealed total resolution of DME and disappearance of HRF in every retinal layer in the central macula. The visual acuity was 20/30, and the CFT was 188 μm at the final visit. (**d**,**e**) A 49-year-old male patient without previous treatment for DME. (**d**) The OCT at baseline exhibited DME with SRF and the presence of HRF in all three retinal layers. The presence of HRF in SRF is highlighted (empty arrow). The baseline visual acuity was 20/100, and the CFT was 859 μm. (**e**) After eight injections in 1 year, the final OCT revealed total resolution of DME and significantly decreased HRF in every retinal layer. The visual acuity was 20/25, and the CFT was 213 μm at the final visit. *CFT* central foveal thickness, *DME* diabetic macular oedema, *HRF* hyperreflective foci, *OCT* optical coherence tomography, *SRF* subretinal fluid.

We collected baseline data including of age, sex, HbA1c at the time of first anti-VEGF treatment, hypertension, lens status, previous treatment (PRP, IVI, steroid, or laser), BCVA in logMAR, fundus photography, fluorescein angiography, and OCT images. The OCT was performed using the RTVue XR Avanti with AngioVue OCTA system (Optovue Inc., Fremont, CA, USA), and fluorescein angiography using Heidelberg retina angiography HRA 2 (Heidelberg Engineering Inc., Germany). The presence of hard exudate on fundus photography, the presence of PDR on fluorescein angiography, CFT, the presence of ERM, intraretinal cysts (IRCs), SRF, and the number of HRF on OCT were recorded. The following data were also collected at 3, 6, and 12 months after first injection: BCVA in logMAR, CFT, number of injections, presence of ERM, IRCs, and SRF, and the number of HRF. HRF were defined as discrete, circumscribed, hyperreflective dots on OCT, with size ranging from 20 to 40 μm (Fig. 3). The location of HRF was classified into three layers, modified from the study by Zur et al.^30^, including inner (from the internal limiting membrane to the inner nuclear layer), outer (from the outer plexiform layer to the ellipsoid zone), and SRF (within the SRF) layers. The amounts of HRF within the central 3 mm of the macula were manually counted by two retinal specialists (TTL and CHH), and the central 3 mm of the macula was defined as 1500 μm on both sides from the foveal centre using the built-in software calliper.

### Literature review

To better understand the role of HRF in predicting the treatment outcomes of DME, we conducted a systematic search using the PubMed database for studies written in any language and published before June 28, 2020. We used the following keywords: (hyperreflective foci) AND (diabetic macular edema). In addition, we reviewed the reference lists of all selected articles to identify other potentially relevant studies. The eligibility criteria were: (1) recruited patients with DME who received any type of treatment; (2) evaluated the presence of HRF, either qualitatively or quantitatively, using OCT; (3) reported on the association of HRF with treatment response of DME, as either final BCVA or BCVA improvement. Two retinal specialists (TTL and CHH) independently reviewed the titles and abstracts of all identified studies and extracted the data from all eligible studies.

### Statistical analysis

For the analysis of BCVA, CFT, and the amount of HRF, the comparisons of measurements between baseline and follow-up visits were performed with paired Student’s t-tests. Linear regression analyses were performed to evaluate the predictive factors for visual outcome and CFT at the 12-month follow-up. The candidate predictive factors included demographic data, OCT biomarkers, drug type, interaction between drug type and HRF, and injection numbers. Age, sex, and pre-treatment logMAR BCVA (for visual improvement) or CFT (for CFT reduction) were adjusted in all models. Linear regression analyses were also performed to evaluate the factors associated with the number of HRF. The candidate predictive factors included age, sex, HbA1c, presence of HTN, treatment history, and presence of PDR or hard exudate. The statistical analysis was performed using SPSS (V.21; IBM Corp., Armonk, NY, USA). A P value lower than 0.05 was considered statistically significant.

## Supplementary Information

Supplementary Information

## Data Availability

The database of current study will be available on request after the evaluation and approval of the request by the Institutional Review Board of National Taiwan University Hospital.
