# Supplementary material for: Hyperreflective foci in predicting the treatment outcomes of diabetic macular oedema after anti-vascular endothelial growth factor therapy
Source: Sci Rep. 2021 Mar 3;11:5103. doi: 10.1038/s41598-021-84553-7 (PMC7930178; doi:10.1038/s41598-021-84553-7)
Supplement: Supplementary file 1 — Supplementary Information [file 41598_2021_84553_MOESM1_ESM.pdf]

Supplemetary Information

Hyperreflective Foci in Predicting the Treatment Outcomes of Diabetic Macular Oedema After Anti-vascular Endothelial Growth Factor Therapy

Chu-Hsuan Huang,[1] Chang-Hao Yang,[2,3] Yi-Ting Hsieh,[2] Chung-May Yang,[2,3] Tzyy-Chang Ho,[2] Tso-Ting Lai [2,4]*

1 Department of Ophthalmology, Cathay General Hospital, Taipei, Taiwan
2 Department of Ophthalmology, National Taiwan University Hospital, Taipei, Taiwan
3 Department of Ophthalmology, College of Medicine, National Taiwan University, Taipei, Taiwan
4 Graduate Institute of Clinical Medicine, College of Medicine, National Taiwan University, Taipei, Taiwan

*Corresponding Author:
Tso-Ting Lai, MD
Department of Ophthalmology, National Taiwan University Hospital, Taipei, Taiwan; Graduate Institute of Clinical Medicine, College of Medicine, National Taiwan University, Taipei, Taiwan
No 7, Chung-Shan S. Rd., Taipei, 100, Taiwan.
Office voice: 886-2-23123456 ext 65205
E-mail: b91401005@ntu.edu.tw

Supplementary Table S1

Predicting factors for best-corrected visual acuity improvement and central foveal thickness reduction at 1 year in patients with diabetic macular oedema under anti-vascular endothelial growth factor therapy.

	VA change at the 12th month		CFT Change at the 12th month	
	P-value	Coefficient	P-value	Coefficient
Age	0.395	0.002	0.096	1.41
Sex (Female =1)	0.524	0.049	0.312	16.17
HbA1c	0.189	-0.034	0.926	-0.660
Hypertension	**0.001**	**0.209**	0.878	2.49
Pseudophakia	0.698	0.027	0.710	-6.83
Previous treatment	**0.031**	**0.136**	0.761	5.32
Proliferative diabetic retinopathy	0.156	0.094	0.919	-1.83
Hard exudate	0.201	-0.074	**0.014**	**-38.18**
Drug type (Aflibercept=1)	0.241	0.070	**0.030***	**34.83**
Baseline VA (LogMAR)	**<0.001**	**-0.337**	0.055	-50.49
Baseline CFT	0.805	0.000067	**<0.001**	**-0.834**
Baseline epiretinal membrane	**0.048**	**0.123**	**0.001**	**54.23**
Baseline intraretinal cyst	0.133	0.126	0.771	6.95
Baseline subretinal fluid	**0.001**	**-0.209**	**0.001**	**-61.33**
Baseline HRF in the inner retina	0.285	-0.018	**0.033**	**-9.69**
Baseline HRF in the outer retina	**0.037**	**-0.007**	**<0.001**	**-4.03**
VA at the 3rd month (LogMAR)	**<0.001**	**0.711**	0.202	-29.37
CFT at the 3rd month	**0.05**	**0.001**	**<0.001**	**0.72**
IVIs at the 3rd month	0.567	-0.042	0.248	-25.35
IVIs at the 6th month	0.419	0.025	0.747	-4.01
IVIs at the 12th month	0.749	-0.005	0.410	-3.47
HRF in the inner retina at the 3rd month	0.645	-0.009	0.493	-3.86
HRF in the outer retina at the 3rd month	**0.024**	**-0.01**	**0.009**	**-3.06**

VA: visual acuity, CFT: central foveal thickness, IVIs: Intravitreal injections, HRF: hyperreflective foci.

*corrected with presence of epiretinal membrane at baseline: p = 0.131
